# Impact of Breakfast Consumption and Sleep Habits on Morning Attention and Concentration Among Health Professional Students

**DOI:** 10.7759/cureus.69592

**Published:** 2024-09-17

**Authors:** Muhammad Abdullah, Khizra Khurram, Aleena Asim, Eshal Naveed, Muhammad Abbas, Hina Zafar Raja, Nasir Saleem, AbdulRahman Fahad Alnaser, Yousef Radhi Aldhafeeri, Fahad Salah Alnusayri

**Affiliations:** 1 Dentistry, Combined Military Hospital (CMH) Lahore Medical College and Institute of Dentistry, Lahore, PAK; 2 Prosthetic Dental Sciences, College of Dentistry, Jouf University, Sakaka, SAU; 3 Prosthodontics, Rahbar College of Dentistry, Lahore, PAK; 4 Operative Dentistry, Rahbar College of Dentistry, Lahore, PAK; 5 Dentistry, College of Dentistry, Jouf University, Sakaka, SAU

**Keywords:** academic performance, breakfast skipping, health students, morning attentiveness, sleep inadequacy, stress reduction

## Abstract

Background

Breakfast consumption and sleep habits are known to affect cognitive performance, yet their specific impact on health professional students' morning attention is underresearched.

Objective

The study's objective was to assess the influence of sleeping habits and breakfast eating habits on health professional students' morning attention span.

Methodology

A cross-sectional study was conducted with 323 undergraduate health professional students from medical colleges in Lahore, Pakistan. Participants completed an online questionnaire that gathered data on demographic characteristics, breakfast consumption patterns, sleep habits, and morning attention levels. Morning attention was measured using a self-reported scale where students rated their ability to concentrate during morning lectures on a five-point Likert scale ranging from "very poor" to "very good." Additional questions assessed the frequency and quality of breakfast consumption, sleep duration, sleep quality (using the Pittsburgh Sleep Quality Index), and daytime napping habits. Statistical analyses, including descriptive statistics and multiple regression analysis, were conducted using the IBM SPSS Statistics for Windows, Version 23 (Released 2015; IBM Corp., Armonk, New York, United States) to explore the relationships between breakfast and sleep habits and their effects on self-reported attention during morning lectures.

Results

Of the 323 participants, 78 (24.14%) never skipped breakfast, while 71 (22.93%) skipped breakfast regularly. Breakfast skippers exhibited poorer attention, with a coefficient of -0.45 (p = 0.0002). Students consuming a more nutritious breakfast demonstrated improved attention, with a coefficient of 0.32 (p < 0.0001). Regarding sleep, 196 students (60.68%) slept four to six hours; those with longer sleep durations had better attention (coefficient = 0.21, p = 0.020). Conversely, 271 participants (83.90%) reported that frequent daytime napping and poor sleep quality, including frequent nighttime awakenings, had a negative impact on attention, with coefficients of -0.30 (p = 0.007) and -0.28 (p = 0.005), respectively. Additionally, 161 students (49.84%) reported difficulty concentrating during morning lectures.

Conclusion

Regular consumption of a nutritious breakfast and sufficient sleep are crucial for maintaining optimal morning attention and cognitive performance among health professional students.

## Introduction

Breakfast is widely regarded as the most important meal of the day, playing a crucial role in providing essential nutrients and energy to support daily functioning. For students, particularly those in health professional fields, proper nutrition is essential not only for maintaining physical well-being but also for enhancing cognitive performance, especially during morning lectures when concentration is most required. Breakfast provides the majority of the day's nutrients and energy, and it is considered an essential meal [1]. Skipping breakfast causes hypoglycemia, followed by a decline in brain function by generating a stress response, which causes reduced learning capability and poor memory retention [2]. Refraining from eating all night long and then skipping breakfast can deprive the nervous system of important nutrients, which can lower productivity and visual attention [3].

It is recommended that each individual get six to seven hours of sleep to enhance their working capacity [4]. Insufficient sleep can cause irritability in behavior and increased susceptibility to diseases, along with other health problems such as high blood pressure and unhealthy weight gain [4]. Troubled sleep cycles were observed among Pakistani medical students in a higher percentage when compared to their non-medical age fellows, according to much research [5]. Moreover, there is a deep relationship between sleep and mental health, which is supported by the fact that good sleep can reduce stress, depression, and anxiety [6,7]. Poor sleep hygiene practices observed in university students include daytime naps, the use of coffee or tea, and the use of smart devices at bedtime [8,9].

In health professional education, attention and concentration are vital for comprehending lectures, excelling in lab work, and delivering high-quality patient care [10]. However, the demanding study schedules of health professional students can adversely affect their sleep and eating habits [11]. Despite the recognized importance of these factors, there is limited research specifically addressing how breakfast consumption and sleep patterns impact the morning attention span of health professional students. Morning attention is the ability to concentrate during morning lectures, which is critical for health professional students to effectively absorb information and perform in both academic and clinical settings. This study aims to fill this research gap by examining the relationship between sleeping habits and breakfast-eating practices and their effects on the concentration and cognitive performance of these students.

Research objective

The study's objective was to assess the influence of sleeping habits and breakfast eating habits on health professional students' morning attention span.

## Materials and methods

Study design and settings

A cross-sectional study was conducted over two years, from July 2022 to August 2024, among undergraduate health professional students from three medical colleges in Lahore, Pakistan. The participating colleges were Combined Military Hospital (CMH) Lahore Medical College and Institute of Dentistry, Akhtar Saeed Medical and Dental College, and Rashid Latif Medical College.

Inclusion and exclusion criteria

The study included undergraduate health professional students from medical colleges in Lahore, Pakistan, aged 18-26 years. Eligible participants were both male and female students who could understand English and provided consent to participate. To ensure relevance to the study's focus on sleep patterns, only students who self-reported having adequate sleep (defined as six to eight hours per night) on the night prior to participation were included. Exclusion criteria were expanded to include students with conditions likely to disrupt sleep or affect concentration, including sleep disorders, depression, obsessive-compulsive disorder (OCD), anxiety disorders, and systemic illnesses such as diabetes. This criterion was established to standardize sleep conditions among participants and to ensure that the study could accurately assess the impact of breakfast habits and sleep patterns on morning attention.

Sample size

A total of 323 students were selected for the study through a convenience sampling method. The sample size was chosen to ensure a representative cross-section of the target population and to achieve statistically significant results. Initial data were collected from 423 students, and a random selection of 323 participants was made to reduce potential biases and enhance the representativeness of the sample.

Data collection

Participants for the study were recruited using a convenience sampling method, targeting undergraduate health professional students from medical colleges in Lahore, Pakistan. Invitations to participate were distributed through institutional email lists, social media groups, and direct invitations during lectures. This approach enabled the rapid collection of data but may have introduced certain biases, such as selection bias, where students who were more accessible or interested in the study might have been more likely to participate.

To mitigate some of these biases, a random selection of 323 participants was made from a total pool of 423 students who initially responded, ensuring a more representative sample for the final analysis. This randomization helped reduce the likelihood of bias while still maintaining the convenience sampling framework.

A structured questionnaire (Appendix 1), administered via Google Forms (Google LLC, Mountain View, California, United States), was used to collect data from the selected participants. The questionnaire comprised 24 questions divided into four sections. The first section gathered demographic details such as age, gender, field of study, year of study, and chronic illnesses. The second section explored participants' breakfast eating habits, including the frequency of breakfast consumption, reasons for skipping breakfast, and typical breakfast foods. The third section assessed participants' sleeping habits and dinner consumption patterns. Sleeping habits were compared to reference values from the Sleep Health Foundation to categorize students as meeting or not meeting recommended sleep quantities. Dinner consumption patterns were also evaluated, including the type and amount of dinner consumed the night before, to examine its potential impact on morning concentration and focus.

The fourth section of the questionnaire evaluated morning attention and concentration during lectures, using items adapted from the Cognitive Failures Questionnaire (CFQ), a validated tool for assessing lapses in attention and concentration. Participants rated their attention levels on a Likert scale from one (very poor) to five (excellent), allowing for quantitative analysis of attention and concentration levels in the morning.

Participants were informed of the study’s purpose, provided consent before completing the questionnaire, and assured of confidentiality, as no personal identifiers were collected.

Validity and reliability

The validity and reliability of the questionnaire were rigorously evaluated to ensure accuracy and consistency. 

Content and Construct Validity

Content validity was established through a rigorous process of expert review. A panel of five professionals with expertise in nutrition, sleep medicine, cognitive psychology, and medical education reviewed the questionnaire. These experts assessed whether the questions adequately covered all relevant aspects of the study’s objectives, specifically, breakfast habits, sleep patterns, and attention-concentration levels. Feedback from these experts was incorporated into the final version of the questionnaire, ensuring that each section was relevant and comprehensive in addressing the study's key variables.

For construct validity, the questionnaire was designed using items adapted from validated instruments, such as the Cognitive Failures Questionnaire (CFQ), for assessing attention and concentration lapses. The CFQ has been extensively validated in prior research for measuring cognitive attention failures, thus providing a strong theoretical foundation for its use in this study. Additionally, sleep patterns were compared against established guidelines from the Sleep Health Foundation, which helped reinforce the construct validity of the sleep-related questions.

Test-Retest Reliability

Test-retest reliability was evaluated by administering the questionnaire to a subset of 30 participants (approximately 9% of the final sample) on two separate occasions, spaced two weeks apart. This interval was chosen to minimize the potential for participants to remember their previous responses while ensuring that their sleep and breakfast habits remained relatively stable. The consistency of responses between the two-time points was analyzed using intraclass correlation coefficients (ICCs), which indicated acceptable levels of stability over time. High ICC values, typically above 0.75, confirmed the reliability of the questionnaire in producing consistent results under similar conditions.

Inter-Rater Reliability

To assess inter-rater reliability, we ensured standardized scoring and interpretation of responses, particularly for subjective questions such as attention-concentration levels. Two independent researchers were trained to apply the scoring rubric consistently. A random sample of 50 questionnaires (around 15% of the total) was evaluated by both researchers. The Cohen’s kappa coefficient was calculated to measure the agreement between the raters. A kappa value above 0.8 indicated strong agreement, demonstrating that the subjective items were reliably scored across different evaluators.

Internal Consistency

Internal consistency was evaluated using Cronbach’s alpha to assess the coherence of items within each section of the questionnaire, particularly those derived from the Cognitive Failures Questionnaire. A Cronbach’s alpha value of 0.7 or higher was considered acceptable, indicating that the items measuring attention and concentration were consistent and related to each other, thereby reinforcing the reliability of the instrument.

Pilot Testing

Finally, face validity was established through a pilot test conducted with a sample of 20 students from the target population who were not included in the final analysis. These participants provided feedback on the clarity, wording, and length of the questionnaire. Based on their input, minor adjustments were made to enhance the clarity and readability of certain questions, ensuring that participants could understand and respond accurately.

Statistical analysis

The collected data were analyzed using IBM SPSS Statistics for Windows, Version 23 (Released 2015; IBM Corp., Armonk, New York, United States). Descriptive statistics were employed to summarize demographic information, including mean, median, standard deviation, and frequency distributions, to provide an overview of the participants' characteristics and their responses to the questionnaire.

Inferential statistics were used to address the research questions and examine the relationships between variables. Correlation analyses, including Pearson correlation coefficients, were conducted to assess the strength and direction of the relationships between breakfast and sleep habits and morning attention-concentration levels to identify significant associations between variables.

To further explore the impact of breakfast and sleep habits on morning attention and concentration, multiple regression analyses were employed for the examination of the relationship between these habits and attention levels while controlling for potential confounders such as age, gender, and field of study. Regression models were constructed with morning attention scores as the dependent variable and breakfast and sleep variables as independent variables.

Subgroup comparisons to determine if the relationships differed by categories such as gender or field of study. Independent sample t-tests and ANOVA were used to compare morning attention scores between groups with different breakfast and sleep patterns.

To assess the robustness of the findings, sensitivity analyses were performed, including re-evaluating the models with different sample sizes and inclusion criteria. Statistical significance was set at a p-value of less than 0.05 for all tests.

The choice of statistical tests was guided by their appropriateness for examining the hypothesized relationships and their ability to control for potential confounders. The use of multiple regression analyses was particularly justified to isolate the effects of breakfast and sleep habits on attention and concentration, ensuring that the findings were not unduly influenced by other variables.

Ethical approval

Ethical approval for the study was obtained from the relevant institutional review board. Participants were provided with an explanation of the study's purpose and methodology, and informed consent was secured prior to data collection. All responses were anonymized to ensure participant confidentiality.

## Results

The distribution of age between male and female students is as follows: 68 (64.76%) of males and 148 (67.89%) of females are in the 18-22 years age group, while 37 (35.24%) of males and 70 (32.11%) of females fall into the 23-26 years age group. The p-value of 0.308 indicates that there is no statistically significant difference in age distribution between male and female students (p > 0.05). This suggests that age does not significantly differ between genders in this study population.

In terms of field of study, 62 (59.05%) male students and 126 (57.89%) female students are enrolled in Bachelor of Medicine and Bachelor of Surgery (MBBS), 30 (28.57%) males and 65 (29.91%) females in Bachelor of Dental Surgery (BDS), and 13 (12.38%) males and 27 (12.20%) females in Allied Health Sciences. The p-value of 0.357 indicates that there is no significant difference in the distribution of fields of study between male and female students (p > 0.05). This implies that gender does not have a significant impact on the choice of field of study among the participants.

Regarding the year of study, 63 (60.00%) male students and 124 (56.88%) female students are in their first year, 31 (29.52%) males and 66 (30.28%) females in their second year, and smaller percentages in the subsequent years. The p-value of 0.786 suggests that there is no significant difference in the distribution across different years of study between male and female students (p > 0.05). This indicates that the distribution of students across different years is similar for both genders.

Chronic illness is reported by 10 (9.52%) male students and eight (3.65%) female students, with the majority of both genders reporting no chronic illness (95 (90.48%) of males and 210 (96.35%) of females). The p-value of 0.212 suggests that there is no significant difference in the prevalence of chronic illness between male and female students (p > 0.05). This indicates that the rate of chronic illness does not significantly differ between genders in this study.

Regarding living arrangements, 63 (60.00%) male students and 118 (54.13%) female students reside in hostels, while 42 (40.00%) males and 100 (45.87%) females are day scholars. The p-value of 0.118 indicates that there is no significant difference in living arrangements between male and female students (p > 0.05). This suggests that the distribution of living arrangements is similar for both genders.

When it comes to breakfast skipping patterns, 27 (25.71%) male students and 51 (23.36%) female students never skip breakfast, 48 (45.71%) of males and 87 (39.91%) of females skip breakfast one to three times a week, 14 (13.33%) of males and 25 (11.46%) of females skip four to six times a week, and 16 (15.24%) of males and 55 (25.23%) of females skip breakfast regularly. The p-value of 0.53 indicates that there is no significant difference in breakfast skipping patterns between male and female students (p > 0.05). This implies that gender does not significantly affect the frequency of skipping breakfast.

The mean sleep duration is 5.52 hours for male students and 5.84 hours for female students. The p-value of 0.099 suggests that there is no significant difference in sleep duration between male and female students (p > 0.05). This indicates that the average amount of sleep is similar for both genders.

The mean attention and concentration score is 3.68 for male students and 3.73 for female students. The p-value of 0.454 suggests that there is no significant difference in attention and concentration scores between male and female students (p > 0.05). This indicates that gender does not significantly affect attention and concentration levels in morning lectures. Overall summary is shown in Table 1.

**Table 1:** Demographic information and statistical comparisons between male and female participants a: chi-square test; b: independent t-test; MBBS: Bachelor of Medicine and Bachelor of Surgery; BDS: Bachelor of Dental Surgery

Parameter	Sub-groups	Male Students	Female Students	Total	p-value
Age group	18-22 Years	68 (64.76%)	148 (67.89%)	216 (66.88%)	0.308^a^
	23-26 Years	37 (35.24%)	70 (32.11%)	107 (33.12%)	
Field of study	MBBS	62 (59.05%)	126 (57.89%)	188 (58.22%)	0.357^a^
	BDS	30 (28.57%)	65 (29.91%)	95 (29.43%)	
	Allied Health Sciences	13 (12.38%)	27 (12.20%)	40 (12.35%)	
Year of study	First Year	63 (60.00%)	124 (56.88%)	187 (57.89%)	0.786^a^
	Second Year	31 (29.52%)	66 (30.28%)	97 (30.05%)	
	Third Year	5 (4.76%)	12 (5.50%)	17 (5.26%)	
	Fourth Year	3 (2.86%)	10 (4.60%)	13 (4.03%)	
	Fifth Year	3 (2.86%)	6 (2.74%)	9 (2.79%)	
Chronic illness	No chronic illness	95 (90.48%)	210 (96.35%)	305 (94.42%)	0.212^a^
	Chronic illness	10 (9.52%)	8 (3.65%)	18 (5.58%)	
Living arrangement	Hostel	63 (60.00%)	118 (54.13%)	181 (56.08%)	0.118^a^
	Day scholar	42 (40.00%)	100 (45.87%)	142 (43.92%)	
Breakfast skipping patterns	Never skipped	27 (25.71%)	51 (23.36%)	78 (24.14%)	0.53^a^
	1-3 times a week	48 (45.71%)	87 (39.91%)	135 (41.85%)	
	4-6 times a week	14 (13.33%)	25 (11.46%)	39 (12.08%)	
	Regularly	16 (15.24%)	55 (25.23%)	71 (22.93%)	
Sleep duration (mean hours)		5.52 hours	5.84 hours	-	0.099^b^
Attention and concentration scores (mean score)		3.68	3.73	-	0.454^b^

Table 2 highlights that 78 participants (24.14%) never skipped breakfast, 135 participants (41.85%) skipped it one to three times a week, 39 participants (12.08%) skipped four to six times a week, and 71 participants (22.93%) skipped regularly. The primary reason for skipping breakfast was waking up late or getting up late, as reported by 160 participants (49.54%), followed by not feeling like eating in the morning (116 participants, 35.92%). Other reasons included disliking available food options (50 participants, 15.48%), having to prepare breakfast themselves (25 participants, 7.75%), feeling nauseous after waking up (35 participants, 10.85%), and concerns about weight (15 participants, 4.65%).

**Table 2:** Breakfast skipping habits and reasons

Variables		Number of Participants (n)	Percentage (%)
Frequency of skipping breakfast	Never	78	24.14
	1-3 times a week	135	41.85
	4-6 times a week	39	12.08
	Regularly	71	22.93
Reasons for skipping breakfast	Getting late/waking up late	160	49.54
	Didn’t feel like eating in the morning	116	35.92
	Didn’t like the food options available	50	15.48
	Had to prepare it themselves	25	7.75
	Feeling nauseous after waking up	35	10.85
	Concerns about weight	15	4.65

Table 3 outlines the types of breakfast foods consumed and the reasons for coffee/tea consumption among participants. For breakfast, 51 participants (15.78%) chose fruits, 43 (13.31%) opted for cereal, 62 (19.19%) had juices or milkshakes, 138 (42.72%) selected local breakfast foods, 19 (5.88%) ate leftover or frozen foods, and 168 (52.01%) preferred bakery items, jams, or breads. Regarding coffee/tea consumption, 125 participants (38.69%) drank it due to personal preference or taste, 109 (33.74%) consumed it to stay awake, and 69 (21.36%) used it to increase focus.

**Table 3:** Breakfast meals and reasons for coffee/tea consumption

Category	Item/Reason	Number of Participants (n)	Percentage (%)
Breakfast meals	Fruits	51	15.78
	Cereal	43	13.31
	Juices/milkshakes	62	19.19
	Local breakfast foods	138	42.72
	Leftover/frozen foods	19	5.88
	Bakery items/jams/breads	168	52.01
Reasons for coffee/tea consumption	Personal preference/taste	125	38.69
	To stay awake	109	33.74
	To increase focus	69	21.36

Table 4 details various aspects of sleep among participants. For sleep duration, 29 participants (8.97%) slept one to three hours, 196 (60.68%) slept four to six hours, 86 (26.62%) slept six to eight hours, and 12 (3.71%) slept more than eight hours. Insomnia was reported by 76 participants (23.52%), while 247 (76.47%) did not experience insomnia. Regarding daytime naps, 271 participants (83.90%) took naps, with 172 (53.25%) napping for one to two hours and 99 (30.65%) napping for more than two hours. In terms of waking up at night, 143 participants (44.27%) never woke up, 147 (45.51%) woke up one to two times, and 33 (10.21%) woke up three or more times. Finally, 164 participants (50.77%) were more active during the day, while 159 (49.22%) were more active during the night.

**Table 4:** Sleep duration, insomnia, daytime naps, waking up at night, and activity level

Characteristic	Item	Number of Participants (n)	Percentage (%)
Sleep duration	1-3 hours	29	8.97
	4-6 hours	196	60.68
	6-8 hours	86	26.62
	More than 8 hours	12	3.71
Insomnia	Insomnia	76	23.52
	No insomnia	247	76.47
Daytime naps	Daytime naps	271	83.90
Duration of daytime naps	1-2 hours	172	53.25
	More than 2 hours	99	30.65
Waking up at night	Never	143	44.27
	1-2 times	147	45.51
	3 or more times	33	10.21
Activity level	More active during the day	164	50.77
	More active during the night	159	49.22

Table 5 presents data on attention and concentration during morning lectures. About 49.84% of participants reported being unable to concentrate, while 50.16% were able to concentrate. In terms of the duration of concentration, 17.02% concentrated for five to 10 minutes, 29.72% for 10-15 minutes, and 53.26% for 15-30 minutes. Regarding recall of what was learned, 8.36% remembered well, 43.35% remembered some, and 48.29% recalled well when self-studied. Class participation ratings revealed that 30.65% had a rating of 0-1, 53.26% had a rating of 2-3, and 16.09% had a rating of 4-5. During lab performances, 52.94% felt hungry or sleepy, while 47.05% experienced dizziness.

**Table 5:** Attention-concentration during morning lectures

Characteristic		Number of Participants (n)	Percentage (%)
Ability to concentrate	Not able to concentrate	161	49.84
	Able to concentrate	162	50.16
Duration of concentration	5-10 minutes	55	17.02
	10-15 minutes	96	29.72
	15-30 minutes	172	53.26
Recall of what was learned	Remembered well	27	8.36
	Remembered some	140	43.35
	Recall well when self-studied	156	48.29
Class participation rating	0-1 rating	99	30.65
	2-3 rating	172	53.26
	4-5 rating	52	16.09
Feelings during lab performances	Hungry/sleepy	171	52.94
	Dizziness during lab demonstrations	152	47.05

Table 6 provides the results of the regression analysis assessing the impact of breakfast and sleep habits on morning attention-concentration levels. The analysis shows that frequent skipping of breakfast has a significant negative effect on attention, with a coefficient of -0.45 (p = 0.0002), indicating poorer concentration. Conversely, consuming a more nutritious breakfast positively affects attention, as evidenced by a coefficient of 0.32 (p < 0.0001). Regarding sleep habits, a longer duration of sleep is positively associated with better attention, with a coefficient of 0.21 (p = 0.020). On the other hand, frequent daytime napping and poor sleep quality (e.g., waking up at night) have significant negative impacts on morning attention, with coefficients of -0.30 (p = 0.007) and -0.28 (p = 0.005), respectively.

**Table 6:** Regression analysis of breakfast and sleep habits on morning attention-concentration levels

Variable		Coefficient	Standard Error	t-value	p-value
Breakfast eating habits	Frequency of skipping breakfast	-0.45	0.12	-3.75	0.0002
	Type of breakfast consumed	0.32	0.08	4.00	<0.0001
Sleep habits	Hours of sleep	0.21	0.09	2.33	0.020
	Frequency of daytime naps	-0.30	0.11	-2.73	0.007
	Quality of sleep (waking up)	-0.28	0.10	-2.80	0.005

## Discussion

This study explored the impact of breakfast consumption and sleeping habits on the morning attention span of health professional students, revealing significant insights into how these factors influence cognitive performance.

Our findings indicate that frequent skipping of breakfast is significantly associated with poorer attention, with a coefficient of -0.45 (p = 0.0002). This result aligns with existing literature demonstrating that missing breakfast adversely affects cognitive functions, including attention and memory [12,13]. Research has consistently shown that skipping breakfast can lead to decreased cognitive performance due to hypoglycemia and reduced energy availability.

Conversely, students who consumed a more nutritious breakfast exhibited better attention, with a positive coefficient of 0.32 (p < 0.0001). This finding is supported by previous studies that a well-balanced breakfast positively impacts cognitive functions [14,15]. Nutrient-rich breakfasts, including fruits, cereal, and other healthy foods, are associated with improved cognitive performance and enhanced attention.

Regarding sleep habits, our analysis found a positive relationship between sleep duration and attention. Students who slept four to six hours had a coefficient of 0.21 (p = 0.020), suggesting better attention compared to those with shorter sleep durations. This is consistent with previous research highlighting the crucial role of adequate sleep in cognitive functions [16,17]. Longer sleep durations are generally linked to better attention and cognitive performance.

However, frequent daytime napping and poor sleep quality were found to negatively impact attention, with coefficients of -0.30 (p = 0.007) and -0.28 (p = 0.005), respectively. These results reflect the detrimental effects of irregular sleep patterns and inadequate sleep hygiene on cognitive performance. Previous studies have shown that frequent daytime naps and disturbances in sleep quality can impair cognitive functions, including attention [18,19].

Overall, these findings underscore the critical role of breakfast consumption and sleep habits in influencing morning attention and concentration. The negative impact of skipping breakfast and poor sleep hygiene on cognitive performance is well documented, and our study adds to this body of evidence, particularly for health professional students. Maintaining healthy eating and sleeping habits is crucial for supporting optimal cognitive function and academic performance.

Study limitations

This study has several limitations, including its reliance on self-reported data, which may be subject to recall bias and inaccuracies. The cross-sectional design limits the ability to establish causality between breakfast and sleep habits and morning attention. Additionally, the sample was restricted to undergraduate health professional students in Lahore, which may not generalize to other populations or geographic areas. Finally, the study did not account for other factors that might influence cognitive performance, such as stress levels, academic workload, or lifestyle variables. These limitations suggest that further research with longitudinal designs and more diverse samples is needed to confirm and expand upon these findings.

## Conclusions

Our study highlights the significant impact of breakfast consumption and sleep habits on the morning attention and concentration of health professional students. Regularly eating a nutritious breakfast was associated with improved attention, while frequent breakfast skipping negatively affected cognitive performance. Additionally, adequate sleep duration positively influenced attention, whereas poor sleep quality and frequent daytime napping were detrimental. These findings underscore the importance of maintaining healthy eating and sleeping practices to enhance cognitive function and academic performance among health professional students.

However, to build on these insights, further research is needed. Longitudinal studies could explore how long-term dietary and sleep habits affect cognitive outcomes over time. Intervention studies could test specific strategies to improve breakfast habits and sleep quality among students. Additionally, research in diverse cultural and geographical contexts could provide a broader understanding of these factors' impact across different populations. Such studies would help in developing targeted strategies and recommendations to support students' academic and cognitive well-being on a larger scale.

## Appendices

Appendix 1 

**Table 7:** Questionnaire

Section	Question	Response Option 1	Response Option 2	Response Option 3	Response Option 4	Response Option 5	Additional Comments
Demographic details	Age	18	19	20	21	22-26	
	Gender	Male	Female	Other			
	Field of study						
	Year of study	1st Year	2nd Year	3rd Year	4th Year	5th Year	
	Do you have any chronic illnesses (e.g., diabetes, heart disease)?	Yes	No				
	If yes, please specify						
Breakfast eating habits	How often do you eat breakfast?	Daily	4-6 times a week	2-3 times a week	Rarely	Never	
	What are your typical breakfast foods?						
	Reasons for skipping breakfast (if applicable)	Lack of time	Not hungry	Dieting	Other (Please Specify)		
Dinner consumption patterns	What type of dinner do you typically have?	Light (e.g., salad, soup)	Moderate (e.g., rice, vegetables)	Heavy (e.g., meat, rich dishes)			
How much do you typically eat for dinner?	Small portion	Moderate portion	Large portion				
Sleeping habits	On average, how many hours do you sleep each night?	Less than 4	4-6 hours	6-8 hours	More than 8		
	Do you frequently take naps during the day?	Yes	No				
	If yes, how long is your average nap?	Less than 30 minutes	30-60 minutes	More than 60 minutes			
	Do you experience difficulty falling asleep or staying asleep?	Never	Rarely	Sometimes	Often	Always	
	How would you rate the quality of your sleep?	Very Poor	Poor	Fair	Good	Excellent	
	Do you feel rested when you wake up in the morning?	Never	Rarely	Sometimes	Often	Always	
Morning attention and concentration	During morning lectures, how often do you find yourself losing concentration?	Never	Rarely	Sometimes	Often	Always	
	Rate your attention and concentration during morning lectures.	1 (Very Poor)	2	3	4	5 (Excellent)	
	How often do you experience lapses in attention or forgetfulness during morning activities?	Never	Rarely	Sometimes	Often	Always	
	Please rate your overall ability to stay focused in the morning.	1 (Very Poor)	2	3	4	5 (Excellent)	
Additional comments	Do you have any other comments or feedback about your breakfast or sleep habits and their effects?						

## References

[1] Ackuaku-Dogbe EM, Abaidoo B (2014). Breakfast eating habits among medical students. Ghana Med J.

[2] Gajre NS, Fernandez S, Balakrishna N, Vazir S (2008). Breakfast eating habit and its influence on attention-concentration, immediate memory and school achievement. Indian pediatrics.

[3] Pollitt E, Mathews R (1998). Breakfast and cognition: an integrative summary. Am J Clin Nutr.

[4] Watson NF, Badr MS, Belenky G (2015). Recommended amount of sleep for a healthy adult: a joint consensus statement of the American Academy of Sleep Medicine and Sleep Research Society. Sleep.

[5] Waqas A, Khan S, Sharif W, Khalid U, Ali A (2015). Association of academic stress with sleeping difficulties in medical students of a Pakistani medical school: a cross sectional survey. PeerJ.

[6] Neculicioiu VS, Colosi IA, Costache C, Sevastre-Berghian A, Clichici S (2022). Time to sleep?—A review of the impact of the COVID-19 pandemic on sleep and mental health. Int J Environ Res Public Health.

[7] João KA, Jesus SN, Carmo C, Pinto P (2018). The impact of sleep quality on the mental health of a non-clinical population. Sleep Med.

[8] Semsarian CR, Rigney G, Cistulli PA, Bin YS (2021). Impact of an online sleep and circadian education program on university students’ sleep knowledge, attitudes, and behaviours. Int J Environ Res Public Health.

[9] Ali RM, Zolezzi M, Awaisu A, Eltorki Y (2023). Sleep quality and sleep hygiene behaviours among university students in Qatar. Int J Gen Med.

[10] Abdel Meguid E, Collins M (2017). Students' perceptions of lecturing approaches: traditional versus interactive teaching. Adv Med Educ Pract.

[11] Roskoden FC, Krüger J, Vogt LJ (2017). Physical activity, energy expenditure, nutritional habits, quality of sleep and stress levels in shift-working health care personnel. PLoS One.

[12] Edefonti V, Rosato V, Parpinel M (2014). The effect of breakfast composition and energy contribution on cognitive and academic performance: a systematic review. Am J Clin Nutr.

[13] Defeyter MA, Russo R (2013). The effect of breakfast cereal consumption on adolescents' cognitive performance and mood. Front Hum Neurosci.

[14] Fagt S, Matthiessen J, Thyregod C, Kørup K, Biltoft-Jensen A (2018). Breakfast in Denmark. Prevalence of consumption, intake of foods, nutrients and dietary quality. A study from the international breakfast research initiative. Nutrients.

[15] Ilmiasih R, Masruroh NL, Ramulia D (2017). Healthy breakfast on learning concentration of junior high school students. InHealth Science International Conference (HSIC).

[16] Lo JC, Groeger JA, Cheng GH, Dijk DJ, Chee MW (2016). Self-reported sleep duration and cognitive performance in older adults: a systematic review and meta-analysis. Sleep Med.

[17] Vetter C, Juda M, Roenneberg T (2012). The influence of internal time, time awake, and sleep duration on cognitive performance in shiftworkers. Chronobiol Int.

[18] Stavitsky K, Neargarder S, Bogdanova Y, McNamara P, Cronin-Golomb A (2012). The impact of sleep quality on cognitive functioning in Parkinson's disease. J Int Neuropsychol Soc.

[19] Pengsuwankasem N, Sittiprapaporn P, Rattanabun W (2023). The effect of short daytime napping on cognitive function, sleep quality, and quality of life in mild cognitive impairment patients. Neurosci Lett.

